# Extended Investigation of Exposure to Respirable Synthetic Amorphous Silica Dust and Its Potential Impact on Non-malignant Respiratory Morbidity

**DOI:** 10.3389/fpubh.2022.801619

**Published:** 2022-05-12

**Authors:** Mei Yong, Peter Morfeld, Robert McCunney

**Affiliations:** ^1^MY EpiConsulting, Duesseldorf, Germany; ^2^Private Scientist, Schwerte, Germany; ^3^Brigham and Women's Hospital, Harvard Medical School Boston, Boston, MA, United States

**Keywords:** respirable synthetic amorphous silica, lung function, epidemiology, modeling, occupational exposure

## Abstract

**Objectives:**

The present analysis aims to study the health impact of an occupational exposure to respirable synthetic amorphous silica (SAS) dusts, based on the available data from the German study.

**Methods:**

The effect of cumulative exposure to respirable SAS dust on respiratory morbidity were investigated in 462 exposed male workers. Multiple exposure assessments was performed anchored by a most recent measurement series. Internal regression models in addition to Monte Carlo-Multi Model were fitted.

**Results:**

An averaged cumulative respirable SAS dust concentration of 6.44 mg/m^3^-years was estimated. Internal regression models suggested a reduction of 8.11 ml (95% confidence interval: 0.49–15.73) in forced vital capacity (FVC) per 1 mg/m^3^-year increase of exposure. But no effect on forced expiratory volume in 1 s (FEV_1_) and the ratio of the parameters FEV_1_/FVC was observed in association with exposure to a respirable fraction of SAS. No adverse effects on the occurrence of respiratory diseases were indicated.

**Conclusion:**

This study provides no clear evidence of adverse health effects from occupational exposure to respirable SAS.

**Sponsor:**

Evonik Operations GmbH/Smart Materials, Cabot Corporation, Wacker Chemie AG.

## Introduction

Pyrogenic and precipitated synthetic amorphous silica (SAS) are nanostructured polymorphs of silicon dioxide, according to the International Organization for Standardization (ISO) definition that “material with any external dimension in the nanoscale or having an internal structure or surface structure in the nanoscale.” Nanoscale is defined as a size range from ~1 to 100 nm. Workers can be exposed to dusts of SAS during production and use of the material. Pyrogenic and precipitated SAS havethe internal structure of primary particles in the nanoscale. However, the aggregate is the smallest divisible entity for an amorphous material like SAS.

Note, Synthetic amorphous silica must be distinguished from crystalline silica. Whereas, adverse health effects from exposure to crystalline silica dust have been identified and studied for decades [e.g., (1)] the situation for chronic SAS dust exposure among humans is quite different. The International Agency for Research on Cancer (IARC) classified crystalline silica as a human lung carcinogen but amorphous silica was categorized into group 3, i.e., “not classifiable as to its carcinogenicity to humans” (2). No cancer risk was linked to SAS dust exposure. Therefore, the major interest in exposure to SAS is the potential for non-malignant respiratory effects. However, the documentation and assessment of published results on respiratory diseases in SAS exposed workers have been insufficient for drawing robust conclusions (3). This situation may be due to the fact that the amorphous forms have never drawn attention given their low toxicity potential (no known specific toxicity, amorphous structure, and solubility of SAS). However, according to a review, risk of respiratory diseases like chronic bronchitis from SAS exposure could not be ruled out (4).

Very few epidemiologic studies that investigated the exposure to amorphous silica and health outcomes are existing. Therefore, the documentation and assessment of published results on respiratory diseases in SAS exposed workers have been insufficient for drawing robust conclusions (3).

A cross-sectional study involving five German SAS production sites has been conducted to investigate the long-term exposure to inhalable SAS dust (5) and the effect on lung function parameters, such as forced vital capacity (FVC), forced expiratory volume in 1 second (FEV_1_), and maximal expiratory flow at 50% of vital flow capacity (MEF50), respiratory diseases, such as chronic bronchitis, and chronic obstructive pulmonary disease (COPD) and pneumoconiosis (abnormalities in chest radiographs) in 484 exposed male workers from five German SAS production plants (6). Average effects of 80 mg/m^3^-years of cumulative inhalable SAS exposure on lung function were estimated (FVC: −48 ml, *p* = 0.04; FEV_1_: −28 ml, *p* = 0.16; FEV_1_/FVC: +0.2%, *p* = 0.39; MEF50: −12 ml, *p* = 0.76). The reduction of FVC was, however, unexceptional when compared to reference values from the non-smoking healthy general population. Hence, no concern for safety and health was substantiated under the existing working condition.

The size of particulate matter has been considered an important determinant of adverse health effects. A thorough review (7) provided a new perspective that the biological activity of SAS can be related to the particle shape and surface characteristics interfacing with the biological milieu rather than to particle size. In epidemiology, the potential health impact from the occupational exposure to SAS, in particular the respirable fraction, is less investigated.

The present study aims to address the potential impact of the respirable fraction of SAS on non-malignant respiratory morbidity with respect to lung function parameters, and prevalence of respiratory pattern, chronic bronchitis, and chronic obstructive pulmonary disease (COPD), we used the available data from the German study involving 462 SAS production workers (5, 6).

## Materials and Methods

### Study Population

The enumerated study population consists of 522 workers exposed to SAS working at five German plants producing either pyrogenic or precipitated SAS (5). The start exposure period of the five German plants varies from 1959 to 1978. Eligible to participate were all current full-time workers in 1997, who worked for at least 1 month at one of the plants. Due to the small number of female workers (about 5%), only male workers were included in the present analysis. Furthermore, 21 workers without exposure information, missing lung function data, missing prick test, and inconsistent data were excluded (6). In total, 462 exposed male workers were eligible for the present analysis. Among the 462 included workers, there are 158 (34%) workers at Plant 1, 29 (6%) at Plant 2, 165 (36%) at Plant 3, 39 (8%) at Plant 4, and 71 (15%) at Plant 5. All study participants gave their informed consent.

### Exposure Assessment

Job-exposure matrices (JEMs) were applied to assess the individual cumulative exposure. Respirable SAS dust concentrations were measured at each of the plants. Within each plant, the same measurement devices were applied, while different teams took the measurement across the plants. The team for Plant 1, 3, and 4 (first team) acted as a trainer for the study teams at Plants 2 and 5. Person-related dust measurements were taken repeatedly and were individually documented. Each participant was measured twice at the first instance and depending on these two measurements a third, fourth, or even fifth measurement was performed. Workers with the same jobs were additionally measured and these measurements were used in later evaluations, although these additional workers were not treated as participants in this study. All measurements were taken in the period from 1997 to 2000 (5). Industrial hygiene and plant experts assessed all jobs across the five plants to create similar exposure groups (SEGs), which means that jobs were rated as one category if the exposure levels in the work environment were comparable (8). The seven different SEGs were categorized as 9 if the categorization was not possible and 0 for the lowest exposure category up to 5 for the highest exposure category. At each plant, changes in production, ventilation, housekeeping, and other factors were reported in detail for each year and SEG since the start of the SAS production. With this information, the experts could assess the exposure levels at each plant by relative scoring (9).

Together with the individual measurement data from the period 1997 to 2000 these relative estimates were anchored to derive the respirable SAS dust concentration estimates for the complete exposure period (10). To do so, a multiple SAS exposure assessment was performed with five calculated statistics to function as anchor values for the individual measurement data. These five statistics were p25 (first quartile), median, p75 (third quartile), the geometric, and the arithmetic mean. Furthermore, uncertainties in relative scoring were assessed by the experts by estimating low, medium, and high relative SAS dust level changes. Routine data from personnel files of the five plants were used to create the individual working history of each worker. With the anchoring method (p25, median, p75, geometric and arithmetic mean) and the type of backward extrapolation (low, medium, high), an array of 15 exposure scenarios were presented as follows, which led to 15 JEMs.

kum1 mean_high scenario

kum2 mean_medium scenario

kum3 mean_low scenario

kum4 p75_high scenario

kum5 p75_medium scenario

kum6 p75_low scenario

kum7 median_high scenario

kum8 median_medium scenario

kum9 median_low scenario

kum10 p25_high scenario

kum11 p25_medium scenario

kum12 p25_low scenario

kum13 geomean_high scenario

kum14 geomean_medium scenario

kum15 geomean_low scenario

This data was then combined with the job histories to constitute an individual exposure profile and across time to derive 15 basic estimates of cumulative exposure to respirable SAS dust for every worker in the study (11, 12). The procedure is described in detail in earlier publications (5, 6).

In analogy to the exposure assessment of inhalable SAS dust (5), the kum8 scenario (median-medium) was based on the medium backward extrapolation estimate anchored at the median of the respirable SAS dust measurements and was less affected by outliers. The kum8 scenario yield the estimates which proved to present the best agreement with the estimates derived from another JEM procedure based on expert judgments. Therefore, it was chosen to serve as the leading exposure scenario when exploring potential health effects due to respirable SAS dust exposure.

### Definition of Health Outcomes

Information regarding demographics, height, occupational history (including prior exposures and co-exposures to hazardous substances), smoking habits, medical history, and current respiratory symptoms were collected with the baseline questionnaires. Interviewers differed between plants and the questionnaires were either interviewer- or self-administered.

The prevalence of respiratory diseases, including chronic bronchitis, chronic obstructive pulmonary disease (COPD), and pneumoconiosis was then determined based on the questionnaires. For chronic bronchitis, the WHO definition of cough and sputum for at least 3 months in at least two consecutive years was applied (13–15). The diagnosis of COPD was based on the GOLD criteria (16, 17). For analysis, two categories, COPD ≥ I and COPD ≥ II, were defined based on lung function data only. Additionally, obstructive chronic bronchitis was defined as COPD ≥ I and COPD ≥ II based on lung function data and the presence of chronic bronchitis. Using poster anterior chest radiographs according to the International Labor Office (ILO) classification 1980, pneumoconiosis was defined as profusion category ≥1/0 (any type: 1/0 or 1/1) by at least one of the three independent readers (18). Atopy was assessed by a skin prick test using four different allergens (cat, grass, birch, and house dust mite). Furthermore, IgE antibodies for several environmental allergens were applied for atopy assessment.

Spirometry was performed by occupational physicians working at the different plants or a technician of the Institute of Preventive and Occupational Medicine (IPA) (at Plant 2). All measurements were either taken with Masterlab® (Plant 3 and Plant 4), portable pneumotachographs (Flowscreen®, Plant 1 and Plant 5), or Masterscope® (Plant 2) to obtain the forced expiratory volume in 1 s (FEV_1_), forced vital capacity (FVC), FEV_1_/FVC ratio, and maximal expiratory flow at 50% of vital flow capacity (MEF50). Each of the devices was produced by the former German company Viasys and used the same pneumotachograph. The measurements were performed according to the recommendations of the American Thoracic Society (19), but FEV_1_ and MEF50 were based on the best FVC maneuver. For each of the three lung function parameters FEV_1_, MEF50, and FVC at least three satisfying forced expiratory maneuvers were done. For Plant 3 and 4, only the best maneuver for FEV_1_, MEF50, and FVC was used.

Afterward, values for FVC, FEV_1_, FEV_1_/FVC ratio, and MEF50 of each worker were expressed as Z-scores according to the reference values according to Quanjer et al. (20) *(European Respiratory Society)*. These Z-scores were then compared to the −1.64 for the lower limit of normality (LLN), i.e., the lower 5% of a normal distribution, to define the respiratory patterns, such as normal (FEV_1_/FVC ≥ LLN and FVC ≥ LLN), obstructive (FEV_1_/FVC < LLN), or restrictive pattern (FEV_1_/FVC ≥ LLN but FVC < LLN) (21).

### Statistical Analyses

The continuous variables of the baseline study characteristics and the spirometry parameters were described with mean ± standard deviation (SD), median and interquartile range (IQR), while the categorical variables were summarized in counts and percentages. Multivariable linear regression models were performed to estimate the effect of a unit increase of cumulative respirable SAS dust exposure (1 mg/m^3^-year) on the lung function parameters (FEV_1_ in ml, FVC in ml, FEV_1_/FVC-ratio in %, MEF50 in ml) by the median-medium scenario (kum8). The regression models included a set of covariates to adjust for potential confounding effects. The same covariates used in the assessment of inhalable SAS dust (6) were included in the models of the present study, to ease comparison between the effect estimates of inhalable and respirable SAS dusts. These covariates were planted effect (Plant 2–5 vs. Plant 1 as reference), age in years, height in cm, body mass index in kg/m^2^, former and current smoker vs. non-smoker, pack-years of smoking corresponding 20 cigarettes per day during 1 year, atopy assessed by skin prick test and IgE antibodies, use of anti-obstructive medication, and prior exposure to fibrogenic dust and substances causing an obstruction. Adjustment for plants was necessary because not only the teams measuring the lung function but also the measurement devices differed across the plants. To correct for heteroscedasticity, robust variance estimates (sandwich estimator) were used in the regression models (22). Multivariable polytomous logistic regression models yielding odds ratios (ORs) were performed on obstructive and restrictive patterns vs. normal spirometry. Same covariates as in the linear regression models were applied, but the smoking status was changed to current smoker vs. former and non-smoker due to missing former smokers for the restrictive pattern. Cumulative respirable SAS dust exposure was expressed as an increase of 1 mg/m^3^-year. Next to the regression models on the respiratory impairment, multivariable logistic regression models on respiratory diseases (chronic bronchitis, obstructive chronic bronchitis, and COPD) and cumulative respirable SAS dust exposure have been conducted. Cumulative SAS exposure was categorized as ≤2, >2– ≤ 6, and above 6 mg/m^3^-year. These categories were based on the distribution, i.e. tertiles (2 mg/m^3^ for 33.33% and 6 mg/m^3^ for 66.66%) for the median-medium scenario. For smoking status, the categories of the current smoker and former smoker vs. non-smoker as in the linear regression models were used. Values of the lung function parameters measured in this study were expressed as a percentage of the Quanjer reference values and skewness and kurtosis have been calculated by the d'Agostino test for each distribution (20). Average respirable SAS dust concentrations (median-medium scenario) for the whole exposure period from 1960 to 2000 were estimated by spline regression and plotted as cubic spline with corresponding 95% confidence intervals for each exposure year. To investigate a potential dose-response relationship between respiratory impairment, respiratory diseases, and cumulative respiratory SAS dust exposure, restricted cubic splines using previous logistic regression models with the 5th, 50th, and 95th percentile as cubic knots have been calculated and plotted. Correlation between the inhalable and respirable SAS dust fraction of the 462 exposed male workers was calculated to determine the strength of the relationship between the two SAS types. Furthermore, this correlation was evaluated separately in each plant to check for plant-specific differences. Due to uncertainties in the anchoring and backward extrapolation for the exposure assessment, Monte Carlo simulations for the linear and logistic regression models as applied above have been performed with 10,000 repetitions for each of the regression models. The Monte Carlo procedure is described in detail previously (6). Fractional polynomials of degree two were used in order to take into account the possible non-linearity in age trends for the association of cumulative respirable SAS dust exposure and the lung function parameters. Additional age effects on the lung function parameters were assessed by calculating the age coefficients of lung function regression analyses according to reference values of the European Respiratory Society (20). These coefficients were compared to the age coefficients in this study for the whole study group of 462 exposed male workers and a subgroup aged 25 years and above.

All analyses were done with Stata 13 (23). Fractional polynomials were fitted with the “fracpoly” command. Monte Carlo regression analyses were performed with the “simulate” command. The statistical significance level was defined at 5%. Adjustment for multiple testing is not considered.

## Results

### Characteristics of the Study Population

Characteristics of the study population are summarized in Table 1. The mean age at baseline was 41 years, the mean duration of exposure was 13.2 years and 59.1% of this study group were active smokers. A total of 52 cases of chronic bronchitis and 69 cases of COPD were counted among the workers. A total of 17 of the 52 cases with chronic bronchitis were additionally classified as obstructive chronic bronchitis. Cases with chronic bronchitis were identified by questionnaire and COPD cases by spirometry criteria only. Furthermore, there were 79 workers with an obstructive respiratory pattern, 16 workers with a restrictive respiratory pattern, and 367 workers with a normal respiratory pattern.

**Table 1:** Study characteristics for 462 SAS-exposed male workers.

**Characteristic**	**Mean (SD)**	**Median (IQR)**	***N* (%)**
Age (baseline) (yrs)	41.0 (9.8)	39.5 (33.6–48.3)	
Height (cm)	176.6 (6.9)	176.0 (172.0–181.0)	
Body mass index (kg/m^2^)	27.2 (4.1)	26.8 (24.4–29.4)	
Duration of exposure (yrs)	13.2 (8.7)	11.6 (7.2–17.4)	
Year of hire	1983 (9.2)	1985 (1977–1989)	
Year or termination	1998 (1.4)	1998 (1996–1998)	
**Smoking**			
Non-smoker			109 (23.6)
Former smoker			80 (17.3)
Smoker			273 (59.1)
Pack-Years of smoking	14.8 (15.2)	12.0 (1.5–20.0)	
**WHO chronic bronchitis**			
Yes			52 (11.3)
No			410 (88.2)
**GOLD obstructive chronic bronchitis (stage)**			
0			445 (96.3)
I			6 (1.3)
II			11 (2.4)
**GOLD spirometric staging for COPD**			
0			393 (85.1)
I			45 (9.7)
II+			24 (5.2)
**Atopy assessment by prick test**			
Positive			163 (35.3)
Negative			299 (64.7)
**Atopy assessment by spec. IgE**			
Positive			148 (32.0)
Negative			314 (68.0)
**Antiobstructive medication**			
Yes			14 (3.0)
No			448 (97.0)
**Prior exposure to fibrogenic dust**			
Yes			77 (16.7)
No			385 (86.3)
**Prior exposure to substances causing obstruction**			
Yes			126 (27.3)
No			336 (72.7)
**Spirometry**			
FEV_1_ (L)	3.9 (0.8)	3.8 (3.4–4.4)	
>LLN			410 (88.7)
≤LLN			52 (11.3)
FVC (L)	5.1 (0.9)	5.0 (4.5–5.7)	
>LLN			436 (94.4)
≤LLN			26 (5.6)
FEV_1_/FVC	0.8 (0.1)	0.8 (0.7–0.8)	
>LLN			394 (85.3)
≤LLN			68 (14.7)
**Respiratory outcome**			
Normal			367 (79.4)
Obstructive pattern			79 (17.1)
Restrictive pattern			16 (3.5)
**Small opacities (radiograph)**			
**Rounded**			
0/0			415 (89.8)
0/1			23 (5.0)
N/A			24 (5.2)
**Irregular**			
0/0			379 (86.5)
0/1			50 (11.4)
1/0			8 (1.8)
1/1			1 (0.2)
N/A			24 (5.2)
**Mixed**			
0/0			435 (94.2)
0/1			3 (0.7)
N/A			24 (5.2)

*LLN: −1.64 according to GLI 2012*.

*N/A, chest radiograph not available; SD, standard deviation*.

### Exposure Assessment of Respirable SAS Dust

The correlation coefficients between the cumulative respirable SAS and inhalable SAS for the median-medium exposure scenario were examined, respectively, on a linear and logarithmic scale.

Strong correlation, ranging from 0.97 to 0.99 for all plants except for plant 2 (0.28) is indicated, with an overall correlation coefficient of 0.87 (*p* < 0.001). To control for the impact of the outlier measurements, the correlations were examined on the logarithmic scale as well. The correlation coefficients are generally improved, for plant 2 (0.60) particularly. An overall correlation coefficient of 0.92 (*p* < 0.001) was yielded.

**Figure 1:**
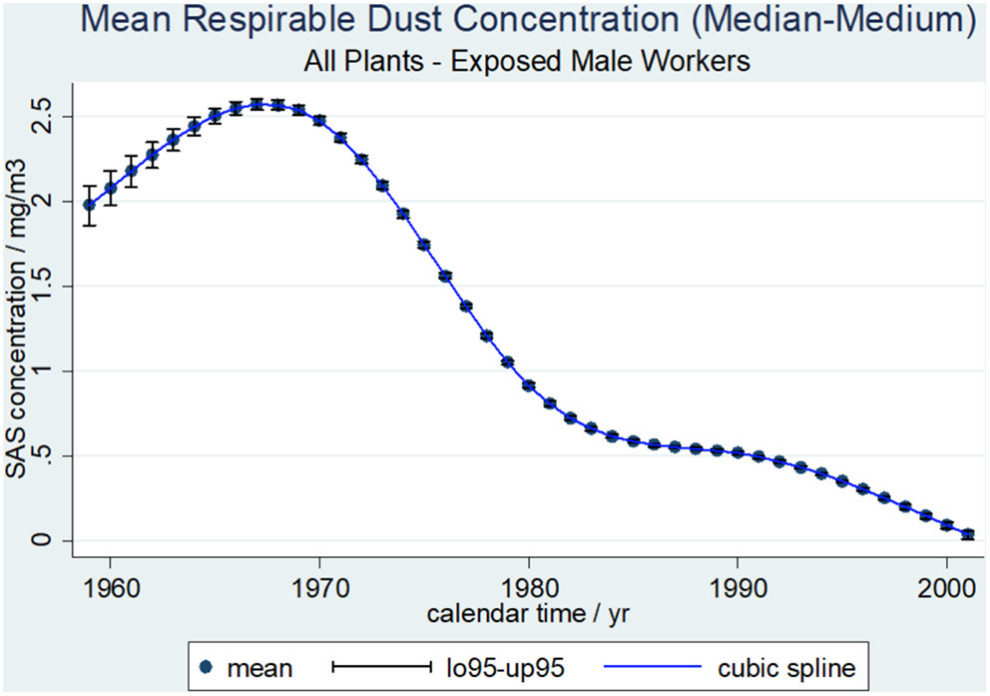
Average respirable SAS dust concentrations (median-medium scenario) and cubic spline function by calendar time with 0.95-confidence limits, 462 SAS exposed male workers.

Average respirable SAS dust concentrations (median-medium scenario) by calendar time with 0.95-confidence limits for all plants and workers are shown in Figure 1. Based on the median-medium scenario the mean SAS concentrations in mg/m^3^ were extrapolated from 1956 (the first working year in the study population) onwards. Calendar time points with a respirable SAS dust concentration below 0 mg/m^3^ were excluded. Until 1966, the mean SAS dust concentration reached its peak value of above 2.5 mg/m^3^ before continuously decreasing to below 0.1 mg/m^3^ in 2001. Even in the job category “bagging” with the highest levels of exposure the median dust concentration was below 0.6 mg/m^3^ at each of the five plants.

Table 2 summarizes the statistics of the cumulative respirable SAS dust concentrations in mg/m^3^-year for the 15 exposure scenarios of all 462 SAS-exposed male workers. Cumulative SAS dust concentrations varied considerably between the 15 exposure scenarios with a mean of 3.45 mg/m^3^-year up to 21.97 mg/m^3^-year. The exposure scenario used in the regression models in this study (median-medium scenario) had an average respirable SAS dust concentration of 6.44 mg/m^3^-year and ranged from 0.2 to 62.7 mg/m^3^-year.

**Table 2:** Distribution of cumulative respirable SAS dust exposures in mg/m^3^-year among the 15 exposure scenarios for 462 SAS-exposed male workers.

**Scenario (*N* = 462)**	**Min**	**P5**	**P25**	**Mean**	**SD**	**P50**	**P75**	**P95**	**Max**
1 Mean-High	0.1242767	0.8621879	2.11227	18.38822	45.02148	5.086327	13.531	71.5805	333.1182
2 Mean-Medium	0.1242767	0.8621879	2.099591	10.63807	16.15442	4.731598	11.61749	38.02087	105.2348
3 Mean-Low	0.0961178	0.8621879	2.099591	8.291332	9.819764	4.453384	10.38533	31.91607	68.39028
4 P75-High	0.1547988	1.0875	2.795124	21.96905	59.1461	5.754957	14.34165	89.49892	439.2065
5 P75-Medium	0.1547988	1.0875	2.718446	11.92538	20.06919	5.591762	11.5534	39.8041	138.5428
6 P75-Low	0.1093381	1.0875	2.713178	8.938291	10.75261	5.536644	10.23994	29.48778	83.22604
7 Median-High	0.1497006	0.536193	1.373239	10.45706	21.562	3.573217	9.571267	38.40878	152.3228
**8 Median-Medium**	**0.1497006**	**0.536193**	**1.340482**	**6.436606**	**8.339037**	**3.470625**	**7.77128**	**23.10788**	**62.66677**
9 Median-Low	0.0948276	0.536193	1.340482	5.133449	5.497449	3.312052	6.694469	16.27296	55.3203
10 P75-High	0.0923077	0.3477631	0.817757	7.280382	16.41187	2.354699	6.293403	30.15792	116.9589
11 P75-Medium	0.0923077	0.3477631	0.798005	4.35689	6.141231	2.246712	4.830932	16.65632	39.48246
12 P75-Low	0.0923077	0.3333333	0.798005	3.450836	3.959503	2.044682	4.068177	12.34656	34.85706
13 Geomean-High	0.1205337	0.6104928	1.54758	12.24693	28.64355	3.756728	9.841978	45.68624	209.8146
14 Geomean-Medium	0.1205337	0.6104928	1.526232	7.146067	10.21232	3.672569	8.125386	23.24833	66.39297
15 Geomean-Low	0.0931322	0.5966656	1.479748	5.568611	6.087117	3.501494	6.899977	18.37606	49.44646

*N, number of subjects; min, minimum; p5, 5th percentile; p25, 25th percentile; mean, arithmetic mean; sd, standard deviation; p50, median; p75, 75th percentile; p95, 95th percentile; max, maximum*.

*The exposure estimates based on median-medium scenario (in bold) are used for the regression analyses*.

### Association Between Cumulative Respirable SAS Dust Exposure and Lung Function Parameters

Valid lung function measurements according to the quality criteria of the American Thoracic Society (19) were collected for FVC, FEV_1_, and FEV_1_/FVC in 462 SAS dust exposed male workers and in 456 SAS dust exposed male workers for MEF50.

#### External Comparisons

**Figure 2:**
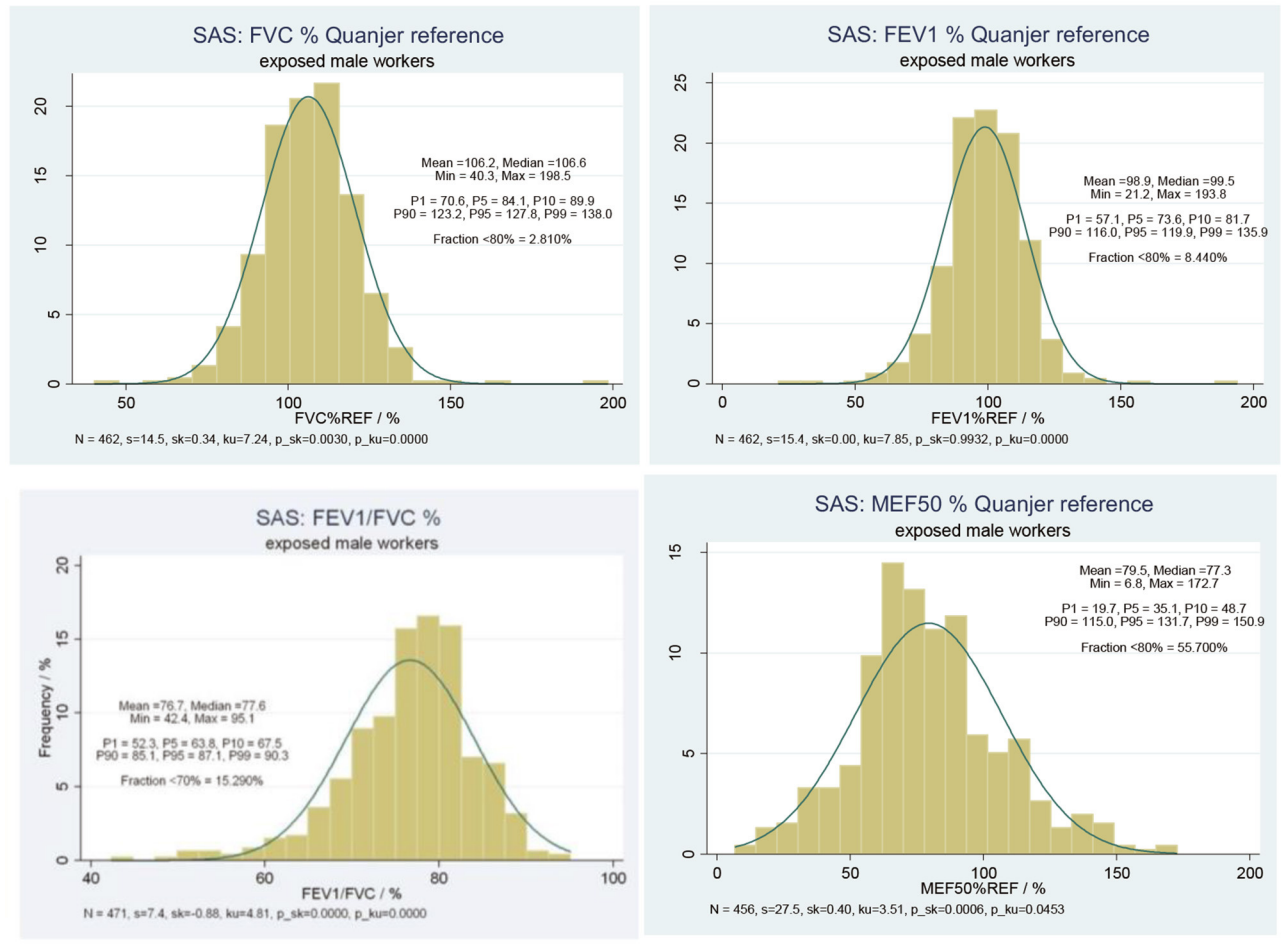
Predictive lung function parameters (i) FVC, (ii) FEV1, (iii) FEV1/FVC, (iv) MEF50, relative to Quanjer reference value among 456 SAS exposed male workers.

Figure 2 displays the results of external comparisons, based on the predictive values of the respective lung function parameters (FVC, FEV_1_, FEV_1_/FVC, MEF50) in relation to the reference values of the European Respiratory Society. Given statistics are mean, median, minimum, maximum, and the percentiles of interest. Additionally, standard deviation (s), skewness (sk), kurtosis (ku), and the *p*-values (p_sk, p_ku) of the d'Agostino test are reported. Furthermore, the fraction of observations below 80% of reference for FVC, FEV_1_, and MEF50 and the fraction of ratios below 70% (16) for the FEV_1_/FVC ratio are shown.

The predictive value of FVC yielded a mean value of ~106% and a median value of about 107%. Only 2.8% of the study population were found with FVC below 80% of the reference value. In parallel, the predictive value of FEV_1_ yielded a mean value of about 99% and a median value ~100%. About 8.4% of the study subjects were found with FEV_1_ below 80% of reference values. On average, a reduction of about 23% in comparison to the reference value was found, 14.5% of all measurements were below 70% of reference. The predictive value of MEF50 yielded a mean value of 79.5% and a median value of 77.3%, which made about 55.7% of all study subjects with MEF50 below 80% of the reference value.

#### Internal Comparisons

For internal comparisons, regression models provide the risk estimate per unit increase of cumulative exposure to respirable SAS, after adjustment for potential confounding factors. The effect estimates with respect to FVC are presented in Table 3. Cumulative exposure to respirable SAS dust (1 mg/m^3^-year) was negatively associated with FVC values with a reduction of 8.11 ml (*p* < 0.05). Apparent heterogeneity across the plants was observed. Compared to plant 1, Plant 3, and Plant 4 presented significantly reduced FVC values (*p* < 0.01). Another plant effect was found in Plant 5, which was positively associated with FVC values (*p* < 0.05). Antiobstructive medication showed a pronounced effect on FVC values in this model, though not reaching statistical significance in this model.

**Table 3:** Multivariable linear regression of FVC and cumulative respirable SAS dust exposure among 462 SAS-exposed male workers by median-medium scenario.

**Response FVC (ml)**	**Linear regression**				
	**Obs** **=** 462; median-medium scenario^*^				
	**Estimate**	**SE**	**Robust 95% CI**		***P*-value**
Intercept	−4,812.16	1,013.29	−6,803.59	−2,820.74	0.000
Cumulative exposure (1 mg/m^3^-year)	−8.11	3.88	−15.73	−0.49	0.037
Plant 2 vs. Plant 1	−96.28	198.76	−486.91	294.35	0.628
Plant 3 vs. Plant 1	−308.25	71.69	−449.15	−167.36	0.000
Plant 4 vs. Plant 1	−294.69	113.57	−517.90	−71.48	0.010
Plant 5 vs. Plant 1	179.52	81.81	18.73	340.31	0.029
Age (years)	−28.10	3.91	−35.79	−20.42	0.000
Height (cm)	64.77	5.18	54.59	74.95	0.000
Body mass index (kg/m^2^)	−8.81	6.84	−22.26	4.64	0.199
Former smoker vs. non-smoker	214.42	97.30	23.20	405.64	0.028
Current smoker vs. non-smoker	−8.29	92.01	−189.12	172.53	0.928
Pack-Years of smoking	−2.96	2.87	−8.61	2.68	0.302
Atopy assessed by prick test (yes/no)	−24.35	73.40	−168.61	119.91	0.740
Atopy assessed by spec. IgE (yes/no)	56.33	72.18	−85.53	198.20	0.436
Antiobstructive medication (yes/no)	−242.18	253.75	−740.88	256.53	0.340
Prior exposure to fibrogenic dust (yes/no)	−92.10	85.66	−260.44	76.25	0.283
Prior exposure to substances causing obstruction (yes/no)	75.98	72.65	−66.79	218.75	0.296

*95% CI, 95% confidence interval*.

* *R^2^ = 0.496*.

Including the same set of covariates, the risks of cumulative exposure to respirable SAS were estimated with respect to FEV1, FEV1/FVC, and MEF50 as well. The risk estimates from the multivariable linear regression of FEV_1_, FVC, FEV_1_/FVC, and MEF50 are summarized in Table 4.

**Table 4:** Overview of effect estimates of cumulative respirable SAS exposure (1 mg/m^3^-year) from respective multivariable linear regression of lung function parameters FVC, FEV_1_, FEV_1_/FVC, and MEF50, among 462 SAS-exposed male workers by median-medium scenario.

	**Linear Regression**				
**Response**	Estimate^*^	**SE**	**Robust 95% CI**		***P*-value**
FEV_1_ (mL)	−5.21	3.43	−11.95	1.52	0.129
FVC (mL)	−8.11	3.88	−15.73	−0.49	0.037
FEV_1_ /FVC (%)	0.03	0.05	−0.06	0.12	0.526
MEF50 (mL)	−3.45	7.69	−18.56	11.67	0.654

*95% CI, 95% confidence interval*.

* *Effect estimates are adjusted for plants (plant 1 as reference), age (years), height (cm), body mass index (kg/m^2^), former smoker vs. non-smoker, current smoker vs. non-smoker, pack-years of smoking, atopy assessed by prick test (yes/no), atopy assessed by spec. IgE (yes/no), antiobstructive medication (yes/no), prior exposure to fibrogenic dust (yes/no), prior exposure to substances causing obstruction (yes/no)*.

With respect to FEV_1_ (effect estimates in ml), the estimated coefficient of cumulative exposure to respirable SAS was not significant (*p* = 0.129). Significant effects were shown regarding the different plants, age, height, smoking, and anti-obstructive medication. Pronounced heterogeneity was observed across the plants. On average, FEV_1_ values decreased by 30.55 ml per year of age (*p* < 0.01) and increased by 44.18 ml per cm increase of body height (*p* < 0.01). Smoking status (former smoker, current smoker vs. non-smoker) and pack years were included in the model at the meantime. Higher pack-years were associated with a decrease in FEV_1_ values by 8.53 ml for each increment of pack-year (*p* < 0.01), while current smoking status was not significant. Former smokers showed a positive association with increased FEV_1_ (*p* < 0.05). A very strong association was indicated from anti-obstructive medication, which was negatively associated with FEV_1_ values (−468.5 ml, *p* < 0.05). BMI, atopy, or prior exposure did not show a statistically significant impact.

In contrast to the models of FVC, no significant effect on the FEV_1_/FVC ratio was observed. Significant effects were found for plants, age, pack-year of smoking, anti-obstructive medication (*p* < 0.01), and height (*p* < 0.05).

With respect to MEF50, neither the cumulative exposure to respirable SAS dust nor the different plants yielded significant estimates, while age, more pack-years of smoking, and anti-obstructive medication were negatively associated with MEF50 (*p* < 0.01).

### Association Between Cumulative Respirable SAS Dust Exposure and Respiratory Pattern, Chronic Bronchitis, and Chronic Obstructive Pulmonary Disease

In Tables 5, 6, the results of the multinomial logistic regression of obstructive (FEV_1_/FVC < LLN) and restrictive patterns (FEV_1_/FVC > LLN and FVC < LLN) compared with normal spirometry (FEV_1_/FVC > LLN and FVC ≥ LLN) according to the Global Lung Initiative (GLI) 2012 are shown (24). Cumulative exposure to respirable SAS dust (1 mg/m^3^-year) for the median-medium scenario did not reach significance for the obstructive pattern, but for the restrictive pattern (OR = 1.07; 95% CI = 1.01–1.12; *p* < 0.05). While for the restrictive pattern no significant odds ratio was found for the other covariates, pack years of smoking (OR = 1.03; 95% CI = 1.00–1.06; *p* < 0.05) and anti-obstructive medication (OR = 8.17; 95% CI = 2.12–31.53; *p* < 0.01) were significantly associated with the obstructive pattern.

**Table 5:** Multivariable logistic regression of WHO chronic bronchitis and cumulative respirable SAS dust exposure among 462 SAS-exposed male workers by median-medium scenario.

**Charactersitic**	**Obs** **=** **462; median-medium scenario**				
	**OR**	**SE**	**Robust 95% CI**		***P*-value**
**Cumulative exposure (mg/m** ^**3**^ **-year)**					
≤2	1.00				
>2– ≤ 6	2.02	0.83	0.90	4.54	0.090
>6	1.03	0.55	0.36	2.93	0.957
**Plants**					
1	1.00				
2	0.10	0.14	0.01	1.44	0.091
3	0.64	0.27	0.28	1.46	0.289
4	0.64	0.37	0.20	2.00	0.440
5	1.22	0.52	0.52	2.82	0.648
Age (years)	1.03	0.03	0.98	1.07	0.299
Height (cm)	0.97	0.01	0.95	0.99	0.000
Body mass index (kg/m^2^)	1.04	0.04	0.97	1.12	0.225
**Smoking**					
Non-smoker	1.00				
Former smoker	0.74	0.52	0.19	2.92	0.664
Current smoker	2.52	1.50	0.79	8.06	0.119
Pack-Years of smoking	1.03	0.01	1.00	1.05	0.064
**Atopy assessed by prick test**					
No	1.00				
Yes	0.89	0.32	0.44	1.81	0.746
**Atopy assessed by spec. IgE**					
No	1.00				
Yes	0.80	0.30	0.38	1.68	0.548
**Antiobstructive medication**					
No	1.00				
Yes	7.40	4.68	2.14	25.56	0.002
**Prior exposure to fibrogenic dust**					
No	1.00				
Yes	1.55	0.76	0.60	4.03	0.369
**Prior exposure to substances causing obstruction**					
No	1.00				
Yes	1.16	0.46	0.53	2.54	0.703

*95% CI, 95% confidence interval; OR, odds ratio*.

**Table 6:** Multivariable logistic regression of chronic obstructive pulmonary disease (COPD) and cumulative respirable SAS dust exposure among 462 SAS-exposed male workers by median-medium scenario.

**Charactersitic**	**Obs** **=** **462; median-medium scenario**				
	**OR**	**SE**	**Robust 95% CI**		***P*-value**
**Cumulative exposure (mg/m** ^**3**^ **-year)**					
≤2	1.00				
>2– ≤ 6	2.45	0.94	1.16	5.20	0.019
>6	1.42	0.68	0.56	3.62	0.462
**Plants**					
1	1.00				
2	0.06	0.59	0.01	0.40	0.003
3	0.61	0.23	0.29	1.28	0.189
4	1.06	0.57	0.37	3.03	0.907
5	0.89	0.38	0.38	2.07	0.792
Age (years)	1.02	0.02	0.98	1.06	0.422
Height (cm)	0.98	0.01	0.97	0.99	0.003
Body mass index (kg/m^2^)	1.00	0.04	0.93	1.08	0.980
**Smoking**					
Non-smoker	1.00				
Former smoker	0.99	0.51	0.36	2.72	0.982
Current smoker	0.75	0.44	0.24	2.35	0.625
Pack-Years of smoking	1.05	0.02	1.02	1.09	0.002
**Atopy assessed by prick test**					
No	1.00				
Yes	0.92	0.31	0.48	1.78	0.810
**Atopy assessed by spec. IgE**					
No	1.00				
Yes	0.87	0.31	0.43	1.74	0.689
**Antiobstructive medication**					
No	1.00				
Yes	4.04	2.48	1.21	13.43	0.023
**Prior exposure to fibrogenic dust**					
No	1.00				
Yes	0.91	0.46	0.34	2.45	0.852
**Prior exposure to substances causing obstruction**					
No	1.00				
Yes	0.85	0.31	0.42	1.72	0.654

*95% CI, 95% confidence interval; OR, odds ratio*.

The multivariable logistic regression of chronic bronchitis according to the WHO criteria and cumulative respirable SAS dust exposure based on the leading exposure estimate (median-medium scenario) among the 462 SAS-exposed male workers is shown in Table 5. Three categories were classified according to the cumulative exposure: ≤2 mg/m^3^-year as the reference category, >2– ≤ 6, and >6 mg/m^3^-year. Neither the cumulative exposure categories (mg/m^3^-year) nor the different plants showed a significant effect on the development of chronic bronchitis. No exposure-response relationship was observed.

Table 6 presents the multivariable logistic regression of COPDand cumulative respirable SAS dust exposure. Compared to the lowest exposure category (≤2 mg/m^3^-year), cumulative exposure to respirable SAS of 2–6 mg/m^3^-year seemed to increase the risk of developing COPD significantly (OR = 2.45; 95% CI = 1.16–5.20), while the highest exposure category (>6 mg/m^3^-year) was not associated with a significantly increased risk (OR = 1.42; 95% CI = 0.56–3.62). No exposure-response relationship could be concluded.

Table 7 summarizes the results of the Monte Carlo regression models on lung function parameters (FEV_1_, FVC, FEV_1_/FVC, MEF50), and the prevalence of respiratory diseases (chronic bronchitis, COPD based on spirometric staging only). The risk estimates of cumulative respirable SAS dust exposure on the lung function parameters were not statistically significant for FEV_1_, FEv1/FVC, and MEF50, while −8.11 (95% CI: −15.71–−0.51) ml decline of FVC was implied. No increased risk of chronic bronchitis and COPD was indicated.

**Table 7:** MC Regression models on the respiratory effects of cumulative exposure among SAS exposed male workers.

	**Precision weighted wean effects given 10,000 MC simulations**			
	**Linear regression**			
**Cumulative exposure) (1 mg/m^**3**^-year)**	**Estimate**	**Robust 95% CI**		
FEV_1_/ml; *N* = 462	−5.21	−11.93	–	1.50
FVC/ml; *N* = 462	−8.11	−15.71	–	−0.51
FEV_1_/FVC/%; *N* = 462	0.03	−0.06	–	0.12
MEF50/ml; *N* = 456	−3.45	−18.52	–	11.63
	**Logistic regression**			
**Cumulative exposure) (1 mg/m^**3**^-year)**	**OR**	**Robust 95% CI**		
**WHO Chronic bronchitis (no/yes); *N* = 462**				
52 cases (11.26%)	1.00	0.96	–	1.04
**GOLD spirometric staging for COPD (stage I, II+)**				
Stage 1; 45 cases (9.7%):	0.98	0.94	–	1.02
Stage II+; 24 cases (5.2%):	1.01	0.95	–	1.08

### Effect of Age on Lung Function Parameters

Table 8 shows the effect of age on lung function parameters according to the reference values of the European Respiratory Society published in the study by Quanjer et al. (20) and additionally the estimated effect in this study for the whole study group and restricted to workers aged over 25 years. In addition to the set of covariates used in the former regression models, an interaction between the different plants and the cumulative exposure was included. The effect estimate of age on FEV_1_ and FVC was comparable in the three groups, while it was greater on MEF50 in the whole study population (−45.89 ml) and those aged above 25 years (−49.73 ml) compared to the males (−31.00 ml) in the study by Quanjer et al. (20). Furthermore, age had a marginally higher impact on the FEV_1_/FVC ratio in the two groups of the present study (−0.17%) than in the Quanjer reference group (−0.13%). Comparing the two groups of the present study, exposed workers aged over 25 seemed to suffer more loss of lung function annually than the whole study population.

**Table 8:** Effect of age on lung function parameters according to reference values of the European respiratory society (20) and estimated in this study.

	**Age coefficients of lung function regression analyses in linear regression**		
	**Quanjer et al**. **(****20****)**	**Exposed males**	**Exposed males**
	**males**	**25+** **y** ***N*** **=** **440**	***N*** **=** **462**
FVC [ml]	−26.01	−28.73	−26.24
FEV_1_ [ml]	−29.03	−30.44	−28.75
FEV_1_/FVC [%]	−0.13	−0.17	−0.17
MEF50 [ml]	−31.00	−49.73	−45.89

## Discussion

The present extended analyses aimed to investigate the effect of cumulative exposure to respirable SAS dust on non-malignant respiratory morbidity. The endpoints are (i) lung function parameters (FEV_1_, FVC, FEV_1_/FVC-ratio, and MEF50); (ii) respiratory pattern (restrictive and obstructive pattern); (iii) respiratory diseases (chronic bronchitis and COPD in 462 exposed male workers of five German plants producing synthetic amorphous silica (SAS). Individual cumulative SAS exposure was calculated by backward extrapolation using 15 different JEMs adapted for uncertainties in historical exposure level estimates. These JEMs were based on anchoring individual working histories; information on changes in production, ventilation, housekeeping, and similar exposure groups (SEGs), and other factors in each plant with the actual SAS measurements in the period of 1997 to 2000.

To our knowledge, apart from the study of Taeger et al. (6) upon which the population of this study was based, only two human studies on occupational SAS exposure and pulmonary function have been performed (25, 26). The cross-sectional study by Wilson et al. consisted of 165 amorphous silica workers. Results showed that pulmonary function and radiographic changes were not significantly associated with neither duration nor total cumulative SAS exposure. In the study of Choudat et al. (25), 41 workers exposed to amorphous silica were compared with an age-matched control group of 90 non-exposed workers at the same plant. Significant differences between exposed and non-exposed workers were found for lung function parameters of forced expiratory forces. No exposure-response relationship was suggested. Both studies suffered from weak exposure assessments, no distinction between inhalable and respirable dust, and no proper adjustments for covariates. The third study reported the adverse health outcome of exposure to inhalable SAS dust (6). To overcome the uncertainties in exposure assessment, tremendous effort was given to check the robustness of exposure estimates, please refer to Morfeld et al. (5). A reduction in FVC, but no effect on FEV_1_ and FEV_1_/FVC was observed in association with exposure to an inhalable fraction of SAS.

The present study reported the evaluation of the health effects of exposure to a respirable fraction of SAS, based on the same study population of Taeger et al. (6). In analog, the leading median-medium scenario among 15 JEMs has been used to estimate cumulative SAS exposure for the present study. Results from the multivariable linear regression models did not indicate any adverse effect on FEV_1_ and FEV_1_/FVC, but a reduction in FVC as a result of exposure to respirable SAS (cf. Table 3).

An estimated loss in FVC of −48 ml resulting from a cumulative exposure of 6.44 mg/m^3^-years needs a discussion. We compared this amount of loss with the loss expected due to aging and smoking. Table 7 shows the age-dependency of lung function parameters according to the reference equations published in Quanjer et al. (20) and it presents the age coefficients estimated in this study for the whole study group and restricted to workers older than 25 years. We used the Quanjer equations to calculate the expected loss in FVC over 40 years: 26 ml/year ^*^40 years = 1,040 ml. Thus, the estimated relative additional loss in FVC due to an exposure to respirable over 40 years was 48/1,040, i.e., <5% (had we used the modeling results, this fraction was slightly smaller). Smokers were found to show a decline in FEV1 of about 60 ml/year (27, 28), equivalent to an additional loss of about 33 ml/year which amounts to an overall additional loss of 1,200 ml over 40 years. Thus, the relative additional loss due to smoking (1,320/1,040) is larger than 100%. Note that a smoker's additional decline in FVC is similar to the smoker's additional decline in FEV1 (29). Thus, we conclude that the additional lung function loss in FVC due to SAS dust exposure as estimated from the MC regression models appears to be negligible when compared to the regular loss due to aging and in particular when compared to the effect of smoking on lung function.

The major weakness of this cross-sectional study is a potential underestimation of exposure and effects since diseased workers might terminate the exposure and not be included in this study population. This so-called “Healthy Worker Survivor Effect” noted as a weakness of Taeger et al. (6) is difficult for the researchers to trace back when the diseased workers have left and the causes for the termination (6). The extensive exposure assessment is the key strength of the present study, using multiple exposure scenarios approach with Job Exposure Matrices as in the present study yields a robust estimate of historical exposure levels. Especially, when assessing historical exposures in different plants and for different job categories, it is important to use an approach that deals with uncertainties in exposure measurements and exposure level changes in the past.

In general, the present cross-sectional study reports that historic workplace exposures to respirable SAS were not associated with respiratory morbidity. However, the effect of the respirable fraction seemed to be more relevant than that of the inhalable fraction, given the identical level of exposure. Since a cross-sectional study has its inherent limitation for causal inference because of the ambiguous temporal order of cause and outcome, a prospective follow-up study with extensive exposure assessment of nanoparticles would provide valid evidence for risk assessment.

## Data Availability Statement

The datasets presented in this article are not readily available because data protection directives.

## Ethics Statement

The studies involving human participants were reviewed and approved by the German Workers Council. The patients/participants provided their written informed consent to participate in this study.

## Author Contributions

PM, MY, and RM: study design, study conduction, and drafting and reviewing of the manuscript. All authors contributed to the article and approved the submitted version.

## Funding

This study was funded by a research contract with Evonik Operations GmbH/Smart Materials, Cabot Corporation, and Wacker Chemie AG. The design, conduct, analysis, and conclusions of the study are exclusively those of the authors.

## Conflict of Interest

MY was employed by MY EpiConsulting. The remaining authors declare that the research was conducted in the absence of any commercial or financial relationships that could be construed as a potential conflict of interest.

## Publisher's Note

All claims expressed in this article are solely those of the authors and do not necessarily represent those of their affiliated organizations, or those of the publisher, the editors and the reviewers. Any product that may be evaluated in this article, or claim that may be made by its manufacturer, is not guaranteed or endorsed by the publisher.
